# Supplementary material for: Laboratory and clinical trials of cocamide diethanolamine lotion against head lice
Source: PeerJ. 2015 Nov 3;3:e1368. doi: 10.7717/peerj.1368 (PMC4636399; doi:10.7717/peerj.1368)
Supplement: Supplemental Information 2 [file peerj-03-1368-s002.pdf]

A randomised, controlled, assessor-blind, parallel group clinical trial to assess the efficacy, safety and acceptability of a surfactant based lotion and a permethrin creme rinse in the treatment of head lice.

DRAFT NUMBER:	4
DATED	31-03-99
PRODUCT NAME:	[Brand name redacted by Sponsor]
COUNTRY:	UK
CLINICAL TRIAL NUMBER:	CT RL01
PRINCIPAL INVESTIGATOR:	Ian F Burgess
CO-INVESTIGATORS:	Christine M Brown
	Dr Nick Irish
ESTIMATED START DATE:	September 1998
ESTIMATED COMPLETION DATE:	December 1998
STUDY MONITOR:	[Name redacted for reasons of privacy]
MEDICAL CONTACTS:	Dr Nick Irish

"Notice: Part or all of the information contained herein may be unpublished material and should be treated as the confidential property of Riemann & Company a/s, not to be divulged to unauthorised persons in any form, including publications and presentations, without the expressed written consent of Riemann & Company a/s.

CONTENTS

Page

1. Introduction 4 1.1
Summary of the Study 4
1.2 Rationale 5
1.3 Aims (Objectives) 7
1.4 Design 7

2. Materials and Methods 7
2.1 Patient Selection 7
2.1.1 Total Numbers of Subjects and Study Duration 7
2.1.2 Inclusion Criteria 7
2.1.3 Exclusion Criteria 8
2.2 Clinical Supplies and Materials 8
2.2.1 Physical Forms of the Study Supplies 8
2.2.2 Packaging and Labelling 8
2.2.3 Care of Supplies 9
2.2.4 Study Materials 9
2.2.5 Compliance 9
2.3 Procedures and Investigations 9
2.3.1 Treatment Regimen/Allocation 9
2.3.2 Randomisation 9
2.3.3 Study Methodology 9
2.3.3.1 Recruitment-Assessment 1 10
2.3.3.2 Further Assessments 10
2.3.3.3 Final Assessment 11
2.3.4 Concomitant Medication 11
2.3.5 Adverse Events 11
2.3.6 Serious Adverse Events 11
2.3.7 Withdrawals 12

3. Analysis and Reports 13
3.1 Definition of End Points 13
3.1.1 Safety 13
3.1.2 Efficacy 13
3.2 Definition of Populations to be analysed 14
3.3 Proposed Primary and Secondary Analyses 14
3.4 Statistical Methods 14
3.4.1 Method of Randomisation 14
3.4.2 Statistical Analysis 14
3.5 Clinical Report 15

4. Administrative Procedures 15
4.1 Regulatory Documentation 15
4.2 Ethics Committee Approval 15
4.3 Informed Consent 15
4.4 Insurance Policy 16

3

CONTENTS (continued)

		Page
4.5	Compensation	16
4.6	Investigator's Responsibilities	16
4.7	Curriculum Vitae	17
4.8	Case Report Form	17
4.9	Monitoring of the Study	18
4.10	Quality Assurance	18
4.11	Protocol Appendices	18
4.12	Protocol Amendments	18
4.13	Publication Policy	18
5.	Investigator's Agreement	19

Appendices

Appendix 1 Letters

Appendix 2 Patient Informed Consent Form and Information Leaflet

Appendix 3 GP letter

1. Introduction

1.1 Summary of the study

Title:	A randomised, controlled, assessor-blind, parallel-group clinical trial to assess the efficacy, safety and acceptability of a surfactant based lotion.
Principal Investigator:	Ian F Burgess
Co-investigators:	Christine M Brown
Estimated Study Dates:	START: September 1998 END: December 1998
Patients/Volunteers:	A total of 120 patients will be recruited to the study. 60 patients will be treated with a surfactant based lotion whilst the remaining 60 will be treated with permethrin creme rinse.
Product:	The surfactant based lotion is a new product called [Brand name redacted by Sponsor]. It contains 10% Cocamide DEA and is widely used in the cosmetic industry. Permethrin creme rinse has been available on the UK market for the treatment of head lice since 1990. It is marketed in the British Isles as Lyclear but known as Nix in the rest of the world.
Method of Applications:	10% Cocamide DEA is applied directly to dry hair and is washed off with shampoo after 60 minutes. Permethrin creme rinse is applied to shampooed and towel dried hair and left in situ for 10 minutes, then rinsed off with clean water.<br><br>Treatments will be applied by experienced investigators throughout the study. Assessments will be made by investigators unaware of the treatment regime used.
Study Design:	The patient will be treated with one of the above products according to a randomisation code. Patients will be assessed at recruitment, (day 0), then days 4, 7, and 10 with a final assessment on day 14. (Recruitment - day 0, followed by 14 days follow-up).
Aims of the Study:	To assess the efficacy and safety of 10% Cocamide DEA and Lyclear/Nix in the eradication of head lice. To assess the ability of each product to kill all viable ova and to assess patient acceptability of the product in use.

1.2 Rationale

Infection with the human head louse (Pediculus humanus capitis) remains of widespread concern in the UK (1,2).

There are many insecticidal compounds available to the patient for the treatment of lice which include carbamates, organophosphates and synthetic pyrethroids (3,4,5,6,7). All these products claim to be ovicidal as well as insecticidal.

Recently, however, two factors have combined to indicate the appropriateness of a formulation capable of killing lice and their eggs but which does not contain a conventional insecticide. The first is the appearance of strains of head lice that have developed resistance to one or more of the currently used insecticides (8,9). The second is an increasing sensitivity on the part of the public to the potential hazards, real or imagined, of exposure to insecticides.

Current alternative therapy for head lice, employing "non-insecticidal" measures such as wet-combing, is time-consuming and dependent upon the skill and motivation of the users (10). It is advantageous to have a treatment that combines a high kill rate of lice with a method that is not time consuming and can be applied easily and effectively. This clinical trial has been designed to evaluate the efficacy of a formulation of a surfactant widely employed in the cosmetic industry. This product can be applied as easily as lotions currently available for treatment of head lice infection and has the added advantage that it does not contain a conventional pesticide and may, therefore, be considered as not damaging to the environment. It will be compared with an accepted insecticide based treatment, Lyclear/Nix containing 1% permethrin.

<u>References</u>

1. Donaldson RJ. The head louse in England: prevalence amongst school children. Health Education Council Report 1975.

2. Department of Health and Social Security, 1988.

3. Sexton C, Miller AJ. A comparison of a single occasion treatment of head louse infestation with phenothrin liquid shampoo or a carbaryl lotion. Curr. Med. Res, Opin. 1991; 12: 466-470.

4. Jolley JH, Kennedy JP, Miller AJ. A comparison of two insecticidal shampoos in the treatment of head louse infestation. J R Soc. Health, 1991; 111: 90-91.

5. Doss S, Powell CA, Miller AJ. Phenothrin lotion, the latest recruit in the battle against head lice: results of two controlled comparative studies. JR Soc. Health, 1991; 111: 47-50.

6. Kyle DR, Comparison of phenothrin shampoo and malathion lotion in the treatment of head louse infection. J R Soc. Health, 1990; 110: 62-63.

7. Burgess IF, Brown CM, Burgess NA. Synergized pyrethrin mousse, a new approach to head lice eradication: efficacy in field and laboratory studies. Clin Ther. 1994; 16: 57-64.

8. Burgess IF, Brown CM, Peock S, Kaufman J. Head lice resistant to pyrethroid insecticides in Britain. Br Med. J, 1995; 311: 752.

9. Burgess I. Management guidelines for lice and scabies. Perscriber, 1996; 7: 87-99.

10. Burgess IF. The dermatoepidemiology of ectoparasitic infections. in: The Challenge of Dermato-epidemiology (HC Williams and DP Stracham, eds) pp 295-311,1997, CRC. Press, Boca Raton.

1.3 Aims (Objectives)

i To evaluate the efficacy of the two products to kill head lice.

ii To evaluate the efficacy of the two products to kill lice eggs - i.e. no small lice noted during follow-up assessments.

iii To monitor the safety of both products in clinical use.

iv To assess the ease of application and removal of each product.

v To assess the overall patient acceptability of each product.

1.4 Design

A total of 120 patients who, following examination, are found to suffer from head lice will be recruited to the trial.

The patient will be treated with the appropriate product according to the randomisation code.

Patients will continue to be assessed on days 4, 7, 10 then again at day 14 after which they will leave the study.

Any adverse events or side effects from the treatments will be monitored during the study.

2. Materials and Methods

2.1 Patient Selection

Selection will be by letters of invitation distributed through participating schools and GP Practices (See appendix 1a).

2.1.1 Total Numbers of Subjects and Study Duration

A total of 120 patients will be recruited to the study. The normal duration of patients in the study will be 15 days (i.e. recruitment - day 0, followed by 14 days follow up assessments).

2.1.2 Inclusion Criteria

1. Male and female patients over the age of 4 who are found to have a head lice infection.

2. Patients who give written informed consent or, if the patient is under 16 years of age, whose guardian gives written informed consent to participate in the study.

3. Available for the duration of study i.e. 15 days.

2.1.3 Exclusion Criteria

1. Patients with a known sensitivity to pyrethroid, organophosphate, and/or carbamate insecticides, sensitivity to chrysanthemums and/or known sensitivity to paraben preservatives.

2. Patients who have been treated with other head lice products within the last 4 weeks.

3. Patients who have undergone a course of antibiotic treatment within the last 4 weeks.

4. Patients who have any persistent skin disorder of the scalp (i.e. eczema, chronic dermatitis, psoriasis).

5. Patients with asthma

6. Patients whose hair has been bleached, colour treated or permed within the last 4 weeks.

7. Pregnant or nursing mothers.

8. Patients who have participated in another clinical trial within 1 month prior to entry to this study.

9. Patients who have already participated in this clinical trial.

2.2 Clinical Supplies and Materials

2.2.1 Physical Forms of the Study Supplies

The test product contains 10% Cocamide DEA in an aqueous emulsion base. Permethrin creme rinse contains 1% 25:75 cis:trans permethrin in a hair conditioner base.

2.2.2 Packaging and Labelling

10% Cocamide DEA is supplied in a polyethylene bottle containing 100 ml and permethrin creme rinse in a polyethylene bottle containing 59ml. All samples will be labelled with appropriate clinical trial labelling.

2.2.3 Care of Supplies

All supplies used in the study must be maintained securely, under the direct responsibility of the principal investigator or under that delegated by the investigator or other personnel licensed to store and dispense drugs.
All supplies shall be dispensed in accordance with the investigator's prescription and it is the investigator's responsibility to ensure an accurate record of supplies, issued and returned, is maintained.

All supplies should be stored at room temperature, out of direct sunlight and protected from humidity and moisture.

2.2.4 Study Materials

All clinical trial materials will be supplied by the Sponsor. Sufficient supplies will be forwarded for the duration of the trial. In addition, case report forms will be supplied for each patient.

2.2.5 Compliance

Treatment will be applied by a member of the investigating team to ensure appropriate use and compliance with instructions. All supplies used, partly used or unused will be maintained for collection by the Study Monitor. Each container of product will be weighed before and after use to measure the quantity of treatment applied and a record of this maintained on the case report form.

2.3 Procedures and Investigations

2.3.1 Treatment Regimen/Allocation

This is a randomised, open, assessor-blind parallel group study of a surfactant based lotion and permethrin creme rinse in the treatment of head lice. All patients who satisfy the inclusion/exclusion criteria will be randomised into one of two groups. One group will be treated with 10% Cocamide DEA, and the second group will be treated with Lyclear/Nix. All treatments will be applied by experienced investigators.

2.3.2 Randomisation

The randomisation code for treatment will be generated by an independent statistician on behalf of Riemann & Company a/s.

2.3.3 Study Methodology

2.3.3.1 Recruitment - Assessment 1

When a patient is thought to be suitable for the study it will be explained to them and/or their guardian. The patient or guardian will be given an information sheet and consent form. Written informed consent must be obtained before the patient can take part in the study.

The following details will then be checked and recorded in the case report form.

1. All inclusion and exclusion criteria will be checked.

2. Patients' initials, sex, date of birth, race, relevant medical history, any concurrent illness and any current medication together with some general information to include how often they wash their hair and what type of shampoo they use, when they last had head lice and what treatment was used.

3. A detection combing will be carried out by the investigator, and those found to have live lice will be invited to join the trial. A record will be made of anyone found to have a heavy infection.

4. Details will be taken of the type of hair: length, thickness, straight or curly etc.

5. Consent will be obtained by signature on the consent form and the patient will then be entered in the trial.

6. Other family members will be inspected for lice if consent is given. Those with lice may also be recruited to the study.

7. The relevant treatment will be applied by the investigator according to the randomisation code and according to the instructions for that particular product. See section 2.3.1 for details of application of the product.

7. The investigators and the volunteers will be asked their opinion of the products used.

8. The patient's GP will be informed.

2.3.3.2 Further Assessments

Patients will not be examined immediately after treatment but the success of the treatments to kill lice and eggs will be made by further assessments undertaken on days 4, 7, 10 with a final assessment on day 14.

At these assessments any lice found will be removed and taped to the Case Report Form, thus rendering the patient non-infective. The patient will remain in the study.

Continued monitoring will enable the investigation to determine whether the presence of lice is due to surviving lice, surviving eggs from which nymphs emerge, or reinfection from contacts. Any patient found to still have lice at day 14 will be offered treatment with an alternative product (Suleo-M lotion).

Any adverse events, or change in concomitant medication will be detailed in the case report form. If necessary any investigator concerns for the patient's welfare will be reported to the Sponsor and a withdrawal form completed if appropriate.

2.3.3.3 Final Assessment

The final assessment will be undertaken 14 days following treatment (day 15 of the study).

Once again if lice are found, they will be removed and taped to the Case Report Form. Doing this allows the investigator to discriminate between re-infection and treatment failure. Re-infection would be indicated by large lice, whereas treatment failure would be indicated by small lice, day 4 onwards, or lice of all stages. Patients who have lice at the end of the study will be offered alternative treatment (Suleo-M lotion).

Any adverse events and changes in concomitant medication will be recorded and at this assessment the completion/withdrawal form will be completed as appropriate.

2.3.4 Concomitant Medication

Patients should not use any other form of pediculicide treatment whilst taking part in this clinical trial. If the use of such treatment occurs, the patient will be withdrawn from the study.

2.3.5 Adverse Events

The case report form will provide space specifically for recording observed and reported adverse events. All unwanted effects**, whether considered to be caused by the study medication or not**, will be reported to the Sponsor by completing the Adverse Events form in the case report form.

2.3.6 Serious Adverse Events

If the adverse event is serious, it shall be reported immediately, by telephone, to the study monitor by the investigators:

TEL: [Telephone number redacted for reasons of privacy]

Serious means fatal, life-threatening, disabling or incapacitating, hospitalisation or prolonged hospitalisation, overdose (of any kind, with or without symptoms), newly diagnosed cancer or clinically abnormal laboratory values (with or without symptoms).

A full written report will then be forwarded to the Sponsor, by fax, within 3 working days.

The contact for all serious adverse events is:

[Monitor name and address redacted for reasons of privacy]

2.3.7 Withdrawals

Patients may be withdrawn from the study at any time for the following reasons:

a) Adverse Event

A patient can be withdrawn from the study because of an adverse event, whether or not the investigator believes it to be serious or caused by the study medication, and provided that the investigator considers it in the patient's best interest to be withdrawn. There must be a corresponding entry on the Adverse Events form in this instance.

b) Non-compliance

The patient is withdrawn because of failure to comply with the investigations as required.

c) Drop Out

The patient withdraws consent to continue in the study, but the investigator would otherwise consider it appropriate for him/her to continue.

d) Lost to Follow-up

The patient, without explanation, fails to be available as scheduled for study assessments and is not seen again despite the investigator's effort (letter, telephone, home visit etc.) to re-establish contact.

e) Death

All deaths will be treated as Serious Adverse Events and the Sponsor must be informed within 24 hours and all associated documentation must be completed within 3 working days. Full details will be required including a post-mortem examination if possible.

f) Treatment failure

The patient is withdrawn by the investigator, or elects to withdraw, because the study medication is not adequately effective and other therapeutic intervention is required.

3. Analysis and Reports

3.1 Definition of End Points

3.1.1 Safety

Patients will be observed and all untoward effects should be recorded, whether or not they are related to the study treatment.
Details of the recording of the adverse events is shown in section 2.3.5 and 2.3.6.

3.1.2 Efficacy

The primary measure is the between treatment comparison of the number of subjects without evidence of active head lice infestation 14 days after enrolment.

"Sample sizes were determined on the basis that one wished to detect as significant at the 95% confidence level equivalence between the two treatment groups to within 20%, assuming that the underlying rates of efficacy would be 90%, and that equivalence would be determined based on confidence limits derived from the normal approximation to the binomial distribution. Since the number of patients required in the efficacy population in each group would be 50 to 80% power and 61 for 90% power, the sample size of 60 per group selected should provide at least 85% power even allowing for possible post-randomization protocol violations (<5%). If in fact the underlying rates of the two treatments averaged 90%, the sample size would have a power of 90% to detect as significant at the 95% confidence level a difference of around 18%".

3.2 Definition of Populations to be analysed

a) The Efficacy Population

This includes all randomised patients who are treated according to study protocol. Premature terminations due to treatment failure, adverse events etc., are also included.

b) "Intention-to-treat" Population

This includes all randomised patients who receive treatment and who have at least one post baseline measurement of efficacy on that treatment. Protocol violators are included in this population. For early discontinuation the last observed values will be carried forward.

3.3 Proposed Primary and Secondary Analyses

See section 3.1.2 for primary effect analysis.

Secondary assessments of efficacy will be:
The safety of the products.
The ease of use.
Patient acceptance.

3.4 Statistical Methods

The statistical analysis will be undertaken by an independent statistician on behalf of the Sponsor.

3.4.1 Method of Randomisation

The two treatments will be allocated to a predetermined randomisation schedule in balanced blocks of 12, prepared by computer-generated random numbers.

3.4.2 Statistical Analysis

The statistical analysis will be undertaken by an independent statistician on behalf of the Sponsor. Differences between groups will be tested based on the "intention to treat" population, while equivalence will be tested based on the "efficacy" population. Equivalence between groups in efficacy will be determined based on 95% confidence limits derived from the normal approximation to the binomial distribution. Differences between groups in efficacy, safety, ease of use and acceptability will be tested using Fisher's exact test and unstratified Chi-squared tests for yes/no variables and the Kruskal-Wallis test for ranked variables. Changes over time will be compared using the Wilcoxon Signed-Ranks test.

3.5 Clinical Report

A Clinical Report of the study, integrating the statistical analyses will be prepared for the study and agreed by the investigator, statistician and the study monitor. A copy of this final report will be forwarded for signature by the principal investigator, the statistician and the study monitor.

4. Administrative Procedures

4.1 Regulatory Documentation

Any required legislative procedures will be undertaken prior to the commencement of the study. The study will not proceed without granted written approval. This study will be conducted according to the recommendations of the European (CPMP) Guidelines on Good Clinical Practice for Trials on Medical Products, and with the European standard, EN 540, 1993; Clinical investigation of medical devices for human subjects.

4.2 Ethics Committee Approval

The investigator will be required to obtain the written approval of his local ethics committee before commencing the study. In accordance with the Good Clinical Research Practice a copy of this will be forwarded to Riemann & Company a/s prior to the release of the study supplies.

4.3 Informed Consent

This study will be conducted in accordance with the principles laid down in the declaration of the World Medical Assembly of Helsinki, as amended in Tokyo, Venice and Hong Kong (see Appendix 3).
Each patient/patient guardian will be requested to give written informed consent after receiving written information and an explanation of what the study involves.

An informed consent form is supplied in the case report form (see Appendix 1). The original consent form will be retained by the investigator, both the investigator and the witness will complete the declaration section of the form. The investigator shall arrange for the retention of patient identification for at least 15 years after the completion or discontinuation of the study.

4.4 Insurance Policy

Riemann & Company a/s confirms that this specific clinical trial is protected by insurance cover which provides an indemnity to the investigators and their co-workers, subject to the Policy terms, conditions and limitations and provided always that the study is conducted and the data as reported agree to the standards fixed by the protocol.

4.5 Compensation

Riemann & Company a/s maintain in force a "no fault" compensation insurance indemnity in accordance with the current version of the ABPI Guidelines on Clinical Trials: "Compensation for Medical Induced Injury ". In the event that the compensation on a "no fault basis" is unacceptable to the claimant, the policy will, subject to its terms, conditions and limitations, respond to an action for legal liability arising out of this clinical trial.

4.6 Investigator's Responsibilities

i. Good Clinical Practice

It is the responsibility of each and every investigator to ensure that this study is carried out in accordance with this protocol in respect of ethical, legal and technical aspects and conforming to both the Declaration of Helsinki and European (CPMP) Guidelines on Good Clinical Practice for Trials on Medicinal Products (see Appendix 4) and also the European standard, EN 540: Clinical investigation of medicinal devices for human subjects (see Appendix 5). In this context, the investigator shall arrange for the retention of patient identification codes for at least 15 years after completion or discontinuation of the trial. The sponsor will render all support necessary to assist the investigator in discharging this responsibility.

ii. Replacement of Investigator

In the event of the investigator being unable to continue the study, another responsible person will be designated investigator and documentation testifying this will be submitted to the study monitor within 10 days. The new investigator must be appropriately qualified and approved by the sponsor and the local ethics committee before the study can be continued.

iii. Study Report

The investigator will submit a summary trial report within approximately 1 month of completion of the study. This report will include:

1. Details of the investigative procedures involved

2. The number of patients entered, completed and withdrawn from the study.

17

3. Deviations from the study protocol on a general basis and for individual patients with explanations.

4. Explanations for each patient withdrawn from the study

5. Methodology and normal ranges for laboratory investigations (where appropriate)

6. Summary of demographic details for each treatment group, e.g. sex, age etc.

7. Summary of the safety and tolerance data, including:

   Details of all ADEs including any follow-up. Case histories of all serious ADEs or ADEs leading to withdrawal.

8. If appropriate, details of any statistical analysis carried out by the investigators, and a summary of efficacy data including clinical observations.

9. Conclusions.

4.7 <u>Curriculum Vitae</u>

In accordance with the international standards, and Good Clinical Research Practice, a signed copy of the curriculum vitae of each investigator and co-worker will be provided to the study monitor.

4.8 <u>Case Report Form</u>

The investigator is required to prepare and maintain adequate and accurate case records which have been designed specifically for this study, and to record all observations and other data pertinent to the clinical investigation. All record forms should be completed in their entirety in a neat, legible manner to ensure accurate interpretation of data. Black-ball-point pen should be used to ensure the clarity of the reproduced copy of all case report forms.

The CPMP Guidelines on Good Clinical Practice for Trials on Medicinal Products in the European Community require that the investigator shall arrange for the retention of the patient identification codes for at least 15 years after the completion or discontinuation of the trial. Patient files and other source data shall also be kept by the Medical Entomology Centre for a period of not less than 15 years.

4.9 <u>Monitoring of the Study</u>

At regular intervals during the study, the study centre may be visited by a representative of the monitoring team of the Sponsor.

4.10 Quality Assurance

In accordance with the Good Clinical Practice Guidelines and recommendations, the Sponsor may undertake a quality assurance audit of the clinical trial and related documentation during the course of this trial.

The purpose of the QA audits is to check on the monitoring of studies and to try to reduce inconsistencies such as transcription errors and errors in logical sequencing. It is important for investigators to maintain an accurate set of patient notes. This is essential material for auditing purposes. At any stage during the trial, the investigator has the responsibility to make all the data available to the sponsor and/or the relevant authority (where required) for auditing purposes. Such audits will at all times be conducted with national, legal and ethical requirements.

4.11 Protocol Appendices

It is specified that the appendices attached to this protocol, and referred to in the main text of this protocol, form an integral part of the protocol.

4.12 Protocol Amendments

No changes or amendments to this protocol can be made by the investigator or by Riemann & Company a/s unless such change(s) or amendment(s) have been fully discussed and agreed upon by both the investigator and Riemann & Company a/s. Any change or amendment agreed upon will be recorded in writing, the written amendment will be signed by the investigator and by Riemann & Company a/s and the signed amendment will be appended to this protocol. A copy of any such changes must be submitted to the Research Ethics Committee for approval before they may be acted upon.

4.13 Publication Policy

Publication results of the study will not take place without prior discussion with Riemann & Company a/s. The company will be allowed sufficient time to analyse these results and provide written agreement to publication. In turn Riemann & Company a/s agree that permission to publish will not be unreasonably withheld. Riemann & Company a/s reserve the right to use the results and reports of this study for their own commercial purposes.

5. Investigator's Agreement

We have read this Riemann & Company a/s approved protocol, number CT RL01, dated June 1997 entitled "A randomised, controlled, assessor-blind, parallel group clinical trial to assess the efficacy, safety and acceptability of a surfactant based lotion and permethrin creme rinse for the treatment of head lice" and have discussed it to our satisfaction with the Sponsor's monitor.

We agree to conduct the study according to this protocol and to comply with its obligations, subject to ethical and safety considerations.

We understand that should we be in the breach of any of the terms of this protocol, or if we are negligent, that Riemann & Company a/s, would not be held responsible for any resulting losses, damages, costs and expenses of whatever kind made by or on behalf of a volunteer.

Principal Investigator :________________________

Co-investigators :________________________

:________________________

[Clinical research Manager] :________________________

[Technical Director] :________________________

Should the decision be made by Riemann & Company a/s to terminate the study at any time, such decision will be communicated to the investigator in writing, and appropriate agreements will be agreed upon and specified in writing. Conversely, should the investigator decide to withdraw from execution of the study he/she will communicate immediately such decision in writing to the Sponsor.

APPENDIX 1a

MEDICAL ENTOMOLOGY CENTRE

Telephones: [number no longer functional]
Fax: [number no longer functional]

Address line 1
Address line 2
Cambridge

Dear Headteacher

The Medical Entomology Centre, in conjunction with Dr N Irish, Consultant in Communicable Disease Control with Cambridge & Huntingdon Health Authority, is embarking on a study to evaluate a potential new way of delivering head louse treatment, which it is hoped will be just as effective as those currently available.

We are planning to recruit 120 patients with head lice for the study and wonder if you would be prepared to distribute information letters to families via your school. School will not be involved administratively in any other way.

If you would like further information please call Mr Ian Burgess or Mrs Christine Brown at the Medical Entomology Centre on [number no longer functional]. We would appreciate a reply as soon as possible as we hope to begin the study during September, with letters going out to families early in the autumn term.

Yours sincerely

Mrs Christine Brown
Medical Entomology Nursing Sister

<u>APPENDIX 1b</u>

MEDICAL ENTOMOLOGY CENTRE

Telephones: [number no longer functional]
Fax: [number no longer functional]

Address line 1
Address line 2
Cambridge

1st July 1998

Dear Parent or Guardian

The Medical Entomology Centre is currently involved in carrying out a field study to assess a new product for the treatment of head lice, and comparing it with a product which has been available in the UK for 6 years.

The new product has been developed in Denmark and is based on a surfactant widely employed in the cosmetic industry. The product can be applied as easily as any currently available lotions used in the treatment of head lice infection but it does not incorporate an insecticide.

If you or anyone in your family has head lice over the next few months perhaps you would consider taking part in this study which will commence immediately. If you are interested in joining the study and have not used a head lice treatment during the previous month please contact either myself or Ian Burgess at the above address as soon as you discover the infection and arrangements will be made to visit you at home. If you contact us by telephone out of office hours leave your name, address, and telephone number (including the STD code) on the answering machine and you will be contacted as soon as possible.

Yours sincerely

Mrs Christine Brown
<u>Medical Entomology Nursing Sister</u>

APPENDIX 2

Patient Information Sheet

Thisw study is designed to test the effectiveness of a new head louse treatment that does not contain a conventional insecticide. The active ingredients in this product are already widely used in a number of cosmetic products. We are comparing the new product with a well known treatment for head lice.

Recently head lice in several parts of Britain and other countries have developed resistance to the insecticides found in some current head lice treatments. The new product should avoid this problem because it works in a different way to kill head lice.

Management of the study will proceed along the following lines:

Volunteers complying with the criteria will be enrolled and coded using randomization. Each person will be checked by one of the investigators using a plastic louse detection comb to confirm that they are infected with head lice. Only one treatment will be applied to each person. If the treatment is with the new product, it will be applied to dry hair, left on for 1 hour and washed off using a conventional toiletry shampoo. If the well known head lice treatment is used it will be applied after washing and towel drying the hair, left on for 10 minutes, then rinsed off using water only.

A different investigator will visit, to comb through the hair to see if any lice remain or eggs have hatched, 4, 7, 10, and 14 days after the treatment. In order for the study to be valid you must be available to the investigator on those days unless there is an unavoidable problem.

During the 14 days after treatment some people may still have a few lice. If any lice are found the will be removed so the person in not infectious to others and no further treatment is required. However, if any lice persist as long as the 14th day examination an alternative treatment will be offered.

You may withdraw from the study at any time and you may then obtain any treatment of your choice outside the study in the normal way without prejudice.

VOLUNTEER AGREEMENT AND CONSENT FORM

1. I, the undersigned, voluntarily agree to participate in a field study using a head louse treatment product.

2. I have been given a full explanation by the supervising professional, of the nature, purpose and likely duration of the study and what I will be expected to do and I have been advised about any discomfort and possible ill-effects on my health or well-being which they believe may result. The information document given to me is attached.

3. I have been given the opportunity to question the supervising professional on all aspects of the study and have understood the advice and information given as a result.

4. I agree to the supervising professional contacting my General Practitioner to make known my participation in the study and I authorise my doctor to disclose details of any relevant medical or drug history, in confidence.

5. I agree to comply with any instruction given during the study and to co-operate faithfully with the supervising professionals and to tell them immediately if I suffer any deterioration of any kind in my health or well-being or any unexpected or unusual symptoms however they may have arisen.

6. I agree that I will not seek to restrict the use to which the results of the study may be put and, in particular, I accept that they may be disclosed to regulatory authorities for medicines in the UK and elsewhere.

7. I understand that I am free to withdraw from the study at any time without needing to justify my decision.

MEDICAL ENTOMOLOGY CENTRE

Consent by a Patient to the participation in a Clinical Trial of a Therapeutic Procedure

I ....

of ....

hereby give my permission fully and freely that I shall participate in the controlled clinical tiral entitled:

A randomised, controlled, assessor-blind, parallel group clinical trial to evaluate the efficacy, safety and acceptability of a surfactant based lotion and permethrin creme rinse in the treatment of head lice.

I understand and acknowledge that the trial is designed to add to medical knowledge and that I may or may not be receiving the most effective available treatment in the first instance.

I note that I may withdraw my consent at any stage in the Investigation and I acknowledge that the purpose of the trial has been explained to me by:

....

and that I have had an opportunity to discuss these matters with him/her.

I have received a written explanation of these matters, a copy of which is attached to this form.

Signed .... Date ....

WITNESS to patient's signature and to the fact that he/she has read the document and freely given his/her consent.

Address:...

Occupation:...

Signed .... Date ....
(Witness must not be a member of project team)

I confirm that I have explained to the patient/volunteer the nature and effect of these procedures.

Signed .... Date ....

(Member of project team acting on behalf of Physician/Surgeon responsible for investigation)

VOLUNTEER AGREEMENT AND CONSENT FORM

1. I, the undersigned, voluntarily agree to my child/children, named in this document, participating in a field study using a head louse treatment product.

2. I have been given a full explanation by the supervising professional, of the nature, purpose and likely duration of the study and what my child/children will be expected to do and I have been advised about any discomfort and possible ill-effects on my child/children's health or well-being which they believe may result. The information document given to me is attached.

3. I have been given the opportunity to question the supervising professional on all aspects of the study and have understood the advice and information given as a result.

4. I agree to the supervising professional contacting my child/children's general practitioner to make known their participation in the study and I authorise my child/children's general practitioner to disclose details of their relevant medical or drug history, in confidence.

5. I agree to comply with any instruction given during the study and to co-operate faithfully with the supervising professionals and to tell them immediately if my child/children suffers any deterioration of any kind in his/her health or well-being or any unexpected or unusual symptoms however they may have arisen.

6. I agree that I will not seek to restrict the use to which the results of the study may be put and, in particular, I accept that they may be disclosed to regulatory authorities for medicines in the UK and elsewhere.

7. I understand that I am free to withdraw my child/children from the study at any time without needing to justify my decision.

Medical Entomology Centre

Consent by Legal Guardian of a Minor to the participation by the Minor in a Clinical Trial of a Therapeutic Procedure.

I ....

of ....

being the legal guardian of ……………………………………………………………………………………………….

of age .... (subsequently referred to as child) hereby give my permission fully and freely for the child to participate in the controlled clinical trial entitled:

A randomised, controlled, assessor-blind, parallel group clinical trial to assess the efficacy, safety and acceptability of a surfactant based lotion and permethrin creme rinse in the treatment of head lice.

I understand and acknowledge that the trial is designed to promote medical knowledge and that my child may or may not be receiving the most effective available treatment in the first instance.

I note that I may withdraw my consent at any stage in the investigation and I acknowledge that the purpose of the trial, has been explained to me by:

....

and that I had an opportunity to discuss these matters with him/her.

I have received a written explanation of these matters, a copy of which is attached to this form.

Signed .... Date ....

WITNESS to guardian's signature and to the fact that he/she has read the document and freely given his/her consent.

Signed .... Date ....

(Witness must not be a member of project team)

I confirm that I have explained to the legal guardian of the child the nature and effect of these procedures.

Signed .... Date ....

Name of Researcher responsible for Investigation ....
(Block capitals)

APPENDIX 3

MEDICAL ENTOMOLOGY CENTRE

Telephones: [number no longer functional]
Fax: [number no longer functional]

Address line 1
Address line 2
Cambridge

To be dated

Dear Dr

The Local Research Ethics Committee has asked us to tell you that the person named below, who is registered with you has agreed to take part in our research project as a volunteer.

Please keep this as a permanent record of their involvement. We shall also inform you later if there are any abnormal findings or possible adverse effects noted by us.

Name of subject	Date of birth	Date(s) of participation in study

Title of Project	Study Reference Number

A randomised, controlled, assessor-blind, parallel-group clinical trial to assess the efficacy, safety and acceptability of a surfactant based lotion and permethrin creme rinse in the treatment of head lice.

Yours sincerely

Mrs Christine Brown
Medical Entomology Nursing Sister
