# Supplementary material for: Laboratory and clinical trials of cocamide diethanolamine lotion against head lice
Source: PeerJ. 2015 Nov 3;3:e1368. doi: 10.7717/peerj.1368 (PMC4636399; doi:10.7717/peerj.1368)
Supplement: Supplemental Information 3 [file peerj-03-1368-s003.pdf]

A randomised, parallel group clinical trial to assess the efficacy, safety and acceptability of a surfactant based lotion in the treatment of head louse infection.

CT RL02

A randomised, parallel group clinical trial to assess the efficacy, safety and acceptability of a surfactant based lotion.

DRAFT NUMBER:	2
DATED	31-03-99
PRODUCT NAME:	[Brand name redacted
COUNTRY:	UK
CLINICAL TRIAL NUMBER:	CT RL02
PRINCIPAL INVESTIGATOR:	Ian F Burgess
CO-INVESTIGATORS:	Christine M Brown
	Dr Nick Irish
ESTIMATED START DATE:	September 1999
ESTIMATED COMPLETION DATE:	March 2000
STUDY MONITOR:	[Name redacted for reasons of privacy]
MEDICAL CONTACTS:	Dr Nick Irish

"Notice: Part or all of the information contained herein may be unpublished material and should be treated as the confidential property of Riemann & Company a/s, not to be divulged to unauthorised persons in any form, including publications and presentations, without the expressed written consent of Riemann & Company a/s.

CONTENTS

		Page
**1.**	**Introduction**	4
1.1	Summary of the Study	4
1.2	Rationale	5
1.3	Aims (Objectives)	7
1.4	Design	7
**2.**	**Materials and Methods**	7
2.1	Patient Selection	7
2.1.1	Total Numbers of Subjects and Study Duration	7
2.1.2	Inclusion Criteria	7
2.1.3	Exclusion Criteria	8
2.2	Clinical Supplies and Materials	8
2.2.1	Physical Forms of the Study Supplies	8
2.2.2	Packaging and Labelling	8
2.2.3	Care of Supplies	9
2.2.4	Study Materials	9
2.2.5	Compliance	9
2.3	Procedures and Investigations	9
2.3.1	Treatment Regiment/Allocation	9
2.3.2	Randomisation	9
2.3.3	Study Methodology	9
2.3.3.1	Recruitment-Assessment 1	10
2.3.3.2	Further Assessments	10
2.3.3.3	Final Assessment	11
2.3.4	Concomitant Medication	11
2.3.5	Adverse Events	11
2.3.6	Serious Adverse Events	11
2.3.7	Withdrawals	12
**3.**	**Analysis and Reports**	13
3.1	Definition of End Points	13
3.1.1	Safety	13
3.1.2	Efficacy	13
3.2	Definition of Populations to be analysed	14
3.3	Proposed Primary and Secondary Analyses	14
3.4	Statistical Methods	14
3.4.1	Method of Randomisation	14
3.4.2	Statistical Analysis	14
3.5	Clinical Report	15
**4.**	**Administrative Procedures**	15
4.1	Regulatory Documentation	15
4.2	Ethics Committee Approval	15
4.3	Informed Consent	15
4.4	Insurance Policy	16

CONTENTS (continued)

		Page
4.5	Compensation	16
4.6	Investigator's Responsibilities	16
4.7	Curriculum Vitae	17
4.8	Case Report Form	17
4.9	Monitoring of the Study	18
4.10	Quality Assurance	18
4.11	Protocol Appendices	18
4.12	Protocol Amendments	18
4.13	Publication Policy	18
**5.**	**Investigator's Agreement**	19

Appendices

Appendix 1 Letters

Appendix 2 Information Document and Consent Forms

Appendix 3 GP Patient Involvement Letter

1. Introduction

1.1 Summary of the study

Title: A randomised, parallel-group clinical trial to assess the efficacy, safety and acceptability of a surfactant based lotion.

Principal Investigator: Ian F Burgess

Co-investigators: Christine M Brown & Dr Nick Irish

Estimated Study Dates: START: September 1999 END: March 2000

Patients/Volunteers: A total of 120 patients will be recruited to the study. 60 patients will be treated once with a surfactant based lotion whilst the remaining 60 will be treated twice, with 7 days between each application.

Product: The surfactant based lotion is a new product called [Brand name redacted by Sponsor]. It contains 10% Cocamide DEA, which is widely used in the cosmetic industry.

Method of Applications: 10% Cocamide DEA is applied directly to dry hair and is washed off at the end of the treatment period.

Method 1 - Apply 10% Cocamide DEA evenly over the hair and scalp. Dry with a hairdryer and leave for 8 hours/overnight prior to washing off with clear water.

Method 2 - Apply 10% Cocamide DEA evenly over the hair and scalp prior to drying with a hairdryer. Wash off with clear water 2 hours after application. Repeat on day 7 (1st treatment day being day 0).

All volunteers will be supplied with a frequent wash shampoo and requested to wash their hair on days 3 and 10 of the study period.

Treatments will be applied by experienced investigators throughout the study. Assessments will be made by investigators unaware of the treatment regime used.

Study Design: The patient will be treated according to a randomisation code. Patients will be assessed at recruitment, (day 0), then days 4, 8, and 11 with a final assessment on day 14. (Recruitment - day 0, followed by 14 days follow-up).

Aims of the Study: To assess the efficacy and safety of 10% Cocamide DEA in the eradication of head lice. To assess the ability of each treatment regime to kill viable ova and to assess patient acceptability of the product.

1.2 Rationale

Infection with the human head louse (Pediculus humanus capitis) remains of widespread concern in the UK (1,2), especially since the appearance of strains of head lice resistant to one or more of the currently available insecticides (3,4).

Whilst there are many insecticidal compounds and formulations available for the treatment of lice (5, 6, 7, 8, 9) which claim to be both ovicidal and insecticidal, alongside the resistance factor there is a growing concern by the public to the potential hazard, real and imagined, of the repeated use of these treatments.

"Non-insecticidal" measures such as wet-combing, whilst time-consuming and dependent on the skill and motivation of the users (10) do not guarantee success, and there is clearly a need for a treatment which combines a high kill rate for both lice and eggs with a method that is easily applied and of a reasonable time span.

Using relatively limited time exposures, an initial clinical study (11) using a surfactant based lotion containing 10% Cocamide DEA, widely employed in the cosmetic industry, showed that whilst some patients were cured, others failed to achieve treatment success evidenced by the continuing presence of mature lice and ovicidal failure.

Similar results were obtained with the comparison treatment, a widely accepted insecticide formulation containing 1% permethrin.

Further laboratory studies varying the time of exposure and using eggs of different ages showed that a longer exposure time kills more eggs and speeding up the rate of drying, by using a conventional hair dryer, concentrates the active ingredient and improves the action.

This clinical trial has been designed to evaluate these findings in the field and to test the efficiency of a repeat treatment one week later, as recommended by the BNF (British National Formulary) for all currently available insecticidal treatments.

References

1. Donaldson RJ. The head louse in England: prevalence amongst school children. Health Education Council Report 1975.

2. Department of Health and Social Security, 1988.

3. Burgess IF, Brown CM, Peock S, Kaufman J. Head lice resistant to pyrethroid insecticides in Britain. Br Med. J, 1995; 311: 752.

4. Sexton C, Miller AJ. A comparison of a single occasion treatment of head louse infestation with phenothrin liquid shampoo or a carbaryl lotion. Curr. Med. Res, Opin. 1991; 12: 466-470.

5. Burgess I. Management guidelines for lice and scabies. Prescriber, 1996; 7: 87-99.

6. Jolley JH, Kennedy JP, Miller AJ. A comparison of two insecticidal shampoos in the treatment of head louse infestation. J R Soc. Health, 1991; 111: 90-91.

7. Doss S, Powell CA, Miller AJ. Phenothrin lotion, the latest recruit in the battle against head lice: results of two controlled comparative studies. JR Soc. Health, 1991; 111: 47-50.

8. Kyle DR, Comparison of phenothrin shampoo and malathion lotion in the treatment of head louse infection. J R Soc. Health, 1990; 110: 62-63.

9. Burgess IF, Brown CM, Burgess NA. Synergized pyrethrin mousse, a new approach to head lice eradication: efficacy in field and laboratory studies. Clin Ther. 1994; 16: 57-64.

10. Burgess IF. The dermatoepidemiology of ectoparasitic infections. in: The Challenge of Dermato-epidemiology (HC Williams and DP Stracham, eds) pp 295-311,1997, CRC. Press, Boca Raton.

11. Report of the findings of Riemann's clinical trial CTRL01.

1.3 Aims (Objectives)

i To evaluate the efficacy of the product to kill head lice.

ii To evaluate the efficacy of the product to kill louse eggs - i.e. no small lice noted during follow-up assessments.

iii To monitor the safety of the product in clinical use.

iv To assess the ease of application and removal of the product.

v To assess the overall patient acceptability of the product.

1.4 Design

A total of 120 patients who, following examination, are found to suffer from head lice will be recruited to the trial.

The patient will be treated using the appropriate regime according to the randomisation code.

Patients will continue to be assessed on days 4, 8, and 11 then again at day 14 after which they will leave the study.

Any adverse events or side effects from the treatment will be monitored during the study.

2. Materials and Methods

2.1 Patient Selection

Selection will be by letters of invitation distributed through schools and GP Practices, and via approved media coverage (See Appendix 1).

2.1.1 Total Numbers of Subjects and Study Duration

A total of 120 patients will be recruited to the study. The normal duration of patients in the study will be 15 days (i.e. recruitment - day 0, followed by 14 days follow up assessments).

2.1.2 Inclusion Criteria

1. Male and female patients over the age of 4 who are found to have a head louse infection.

2. Patients who give written informed consent and, if the patient is under 16 years of age, whose guardian gives written informed consent to participate in the study.

3. Available for the duration of study i.e. 15 days.

2.1.3 Exclusion Criteria

1. Patients with a known sensitivity to paraben preservatives.

2. Patients who have been treated with other head lice products within the last 2 weeks.

3. Patients who have undergone a course of antibiotic treatment within the last 4 weeks.

4. Patients who have any persistent skin disorder of the scalp (i.e. eczema, chronic dermatitis, psoriasis).

5. Patients whose hair has been bleached, colour treated or permed within the last 4 weeks.

6. Patients who have participated in another clinical trial within 1 month prior to entry to this study.

7. Patients who have already participated in this clinical trial.

2.2 Clinical Supplies and Materials

2.2.1 Physical Forms of the Study Supplies

[Band name redacted] contains 10% Cocamide DEA in an aqueous emulsion base.

2.2.2 Packaging and Labelling

10% Cocamide DEA is supplied in a polyethylene bottle containing 100 ml. All samples will be labelled with appropriate clinical trial labelling.
Boots Frequent Wash Shampoo will be supplied in a 50 ml. plastic bottle.

2.2.3 Care of Supplies

All supplies used in the study must be maintained securely, under the direct responsibility of the principal investigator or under that delegated by the investigator or other personnel licensed to store and dispense drugs.

All supplies shall be dispensed in accordance with the investigator's prescription and it is the investigator's responsibility to ensure an accurate record of supplies, issued and returned, is maintained.

All supplies should be stored at room temperature, out of direct sunlight and protected from humidity and moisture.

2.2.4 Study Materials

All clinical trial materials will be supplied by the Sponsor. Sufficient supplies will be forwarded for the duration of the trial. In addition, case report forms will be supplied for each patient.

2.2.5 Compliance

Treatment will be applied by a member of the investigating team to ensure appropriate use and compliance with instructions. All supplies used, partly used or unused will be maintained for collection by the Study Monitor. Each container of product will be weighed before and after use to measure the quantity of treatment applied and a record of this maintained under separate cover. (Appendix 2).

2.3 Procedures and Investigations

2.3.1 Treatment Regimen/Allocation

This is a randomised, parallel group study of a surfactant based lotion. All patients who satisfy the inclusion/exclusion criteria will be randomised into one of two groups. All treatments will be applied by experienced investigators.

Group 1 - 10% Cocamide DEA will be applied to the hair and scalp and the product dried with a hairdryer. It will be washed off with clear water after a minimum of 8 hours or left overnight.

Group 2 - 10% Cocamide DEA will be applied to the hair and scalp and the hair dried with a hairdryer. The product will be washed off with clear water after 2 hours. This treatment regime will be repeated on day 7. (The first treatment day being day 0)

Each patient will be issued with a toiletry shampoo and requested to wash their hair on days 3 and 10. They will be asked not to shampoo their hair on any other day.

2.3.2 Randomisation

The randomisation code for treatment will be generated by an independent statistician on behalf of Riemann & Company a/s.

2.3.3 Study Methodology

2.3.3.1 Recruitment - Assessment 1

When a patient is thought to be suitable for the study it will be explained to them and/or their guardian. The patient or guardian will be given an information sheet and consent form. Written informed consent must be obtained before the patient can take part in the study, this consent to be independently witnessed. (Appendix 3).

The following details will then be checked and recorded in the case report form.

1. All inclusion and exclusion criteria will be checked.

2. Patients' initials, sex, date of birth, relevant medical history, any concurrent illness and any current medication together with some general information to include how often they wash their hair and what type of shampoo they use, when they last had head lice and what treatment was used.

3. A detection combing will be carried out by the investigator, and those found to have live lice will be invited to join the trial . A record will be made of anyone found to have a heavy infection.

4. Details will be taken of the type of hair: length, thickness, straight or curly etc.

5. Other family members will be inspected for lice if consent is given. Those with lice may also be recruited to the study.

6. The relevant treatment regime will be assigned and carried out by the investigator according to the randomisation code and according to the instructions for that particular method. (See section 2.3.1 for details of application of the product).

7. The investigators and the volunteers will be asked their opinion of 10% Cocamide DEA.

8. The patient's GP will be informed. (Appendix 4).

2.3.3.2 <u>Further Assessments</u>

Patients will not be examined immediately after the first treatment but the success of the two regimes to kill lice and eggs will be monitored by further assessments undertaken on days 4, 8, 11 with a final assessment on day 14.

At these assessments any lice found will be removed and taped to the Case Report Form. The patient will remain in the study.

Continued monitoring will enable the investigation to determine whether the presence of lice is due to surviving lice, surviving eggs from which nymphs emerge, or reinfection from contacts. Any patient found to still have lice at day 14 will be offered treatment with an alternative product (Suleo-M lotion).

Any adverse events, or change in concomitant medication will be detailed in the case report form. If necessary any investigator concerns for the patient's welfare will be reported to the Sponsor and a withdrawal form completed if appropriate.

2.3.3.3 <u>Final Assessment</u>

The final assessment will be undertaken 14 days following treatment (day 15 of the study).

Once again if lice are found, they will be removed and taped to the Case Report Form. Doing this allows the investigator to discriminate between re-infection and treatment

failure. Re-infection would be indicated by large lice, whereas treatment failure would be indicated by small lice, day 4 onwards, or lice of all stages. Patients who have lice at the end of the study will be offered alternative treatment (Suleo-M lotion).

Any adverse events and changes in concomitant medication will be recorded and at this assessment the completion/withdrawal form will be completed as appropriate.

2.3.4 Concomitant Medication

Patients should not use any other form of pediculicide treatment whilst taking part in this clinical trial. If the use of such treatment occurs, the patient will be withdrawn from the study.

2.3.5 Adverse Events

The case report form will provide space specifically for recording observed and reported adverse events. All unwanted effects, **whether considered to be caused by the study medication or not,** will be reported to the Sponsor by completing the Adverse Events form in the case report form.

2.3.6 Serious Adverse Events

If the adverse event is serious, it shall be reported immediately, by telephone, or fax to the study monitor by the investigators:

TEL: [Telephone number redacted for reasons of privacy]

Serious means fatal, life-threatening, disabling or incapacitating, hospitalisation or prolonged hospitalisation, overdose (of any kind, with or without symptoms), newly diagnosed cancer or clinically abnormal laboratory values (with or without symptoms).

A **full written report** will then be forwarded to the Sponsor, by fax, **within 3 working days.**

The contact for all serious adverse events is:

[Monitor name and address redacted for reasons of privacy]

2.3.7 Withdrawals

Patients may be withdrawn from the study at any time for the following reasons:

a) Adverse Event

A patient can be withdrawn from the study because of an adverse event, if the investigator considers it to be in the patient's best interest. An Adverse Events form must be filled in whether or not the investigator believes it to be serious or caused by the study medication.

b) Non-compliance

The patient is withdrawn because of failure to comply with the investigations as required.

c) Drop Out

The patient withdraws consent to continue in the study, but the investigator would otherwise consider it appropriate for him/her to continue.

d) Lost to Follow-up

The patient, without explanation, fails to be available as scheduled for study assessments and is not seen again despite the investigator's effort (letter, telephone, home visit etc.) to re-establish contact.

e) Death

All deaths will be treated as Serious Adverse Events and the Sponsor must be informed within 24 hours and all associated documentation must be completed within 3 working days. Full details will be required including a post-mortem examination if possible.

f) Treatment failure

The patient is withdrawn by the investigator, or elects to withdraw, because the study medication is not adequately effective and other therapeutic intervention is required.

3. Analysis and Reports

3.1 Definition of End Points

3.1.1 Safety

Patients will be observed and all untoward effects should be recorded, whether or not they are related to the study treatment.

Details of the recording of the adverse events is shown in section 2.3.5 and 2.3.6.

3.1.2 Efficacy

The primary measure is the between treatment comparison of the number of subjects without evidence of active head lice infestation 14 days after enrolment.

"Sample sizes were determined on the basis that one wished to detect as significant at the 95% confidence level equivalence between the two treatment groups to within 20%, assuming that the underlying rates of efficacy would be 90% power, and that equivalence would be determined based on confidence limits derived from the normal approximation to the binomial distribution. Since the number of patients required in the efficacy population in each group would be 50 to 80% power and 61 for 90% power, the sample size of 60 per group selected should provide at least 85% power even allowing for

possible post-randomization protocol violations (<5%). If in fact the underlying rates of the two treatments averaged 90%, the sample size would have a power of 90% to detect as significant at the 95% confidence level a difference of around 18%".

3.2 Definition of Populations to be analysed

a) The Efficacy Population

This includes all randomised patients who are treated according to study protocol. Premature terminations due to treatment failure, adverse events etc., are also included.

b) "Intention-to-treat" Population

This includes all randomised patients who receive treatment and who have at least one post baseline measurement of efficacy on that treatment. Protocol violators are included in this population. For early discontinuation the last observed values will be carried forward.

3.3 Proposed Primary and Secondary Analyses

See section 3.1.2 for primary effect analysis.

Secondary assessments of efficacy will be:
The safety of the product.
The ease of use.
Patient acceptance.

3.4 Statistical Methods

The statistical analysis will be undertaken by an independent statistician on behalf of the Sponsor.

3.4.1 Method of Randomisation

The two treatment regimes will be allocated to a predetermined randomisation schedule in balanced blocks of 12, prepared by computer-generated random numbers.

3.4.2 Statistical Analysis

The statistical analysis will be undertaken by an independent statistician on behalf of the Sponsor. Differences between groups will be tested based on the "intention to treat" population, while equivalence will be tested based on the "efficacy" population. Equivalence between groups in efficacy will be determined based on 95% confidence limits derived from the normal approximation to the binomial distribution. Differences between groups in efficacy, safety, ease of use and acceptability will be tested using Fisher's exact test and unstratified Chi-squared tests for yes/no variables and the Kruskal-

Wallis test for ranked variables. Changes over time will be compared using the Wilcoxon Signed-Ranks test.

3.5 Clinical Report

A Clinical Report of the study, integrating the statistical analyses will be prepared for the study and agreed by the investigator, statistician and the study monitor. A copy of this final report will be forwarded for signature by the principal investigator, the statistician and the study monitor.

4. Administrative Procedures

4.1 Regulatory Documentation

Any required legislative procedures will be undertaken prior to the commencement of the study. The study will not proceed without granted written approval. This study will be conducted according to the World Medical Association Declaration of Helsinki, and the recommendations of the European (CPMP) Guidelines on Good Clinical Practice for Trials on Medical Products, and with the European standard, EN 540, 1993; Clinical investigation of medical devices for human subjects.

4.2 Ethics Committee Approval

The investigator will be required to obtain the written approval of the local ethics committee in the areas where the study will take place prior to its commencement. In accordance with the Good Clinical Research Practice a copy of this will be forwarded to Riemann & Company a/s prior to the release of the study supplies.

4.3 Informed Consent

This study will be conducted in accordance with the principles laid down in the declaration of the World Medical Assembly of Helsinki, as amended in Tokyo, Venice and Hong Kong (see Appendix 6). Each patient/patient guardian will be requested to give written informed consent after receiving written information and an explanation of what the study involves.

An informed consent form is supplied in the case report form (see Appendix 5). The original consent form will be retained by the investigator, both the investigator and the witness will complete the declaration section of the form. The investigator shall arrange for the retention of patient identification for at least 15 years after the completion or discontinuation of the study.

4.4 Insurance Policy

Riemann & Company a/s confirms that this specific clinical trial is protected by insurance cover which provides an indemnity to the investigators and their co-workers, subject to the Policy terms, conditions and limitations and provided always that the study is conducted and the data as reported agree to the standards fixed by the protocol.

4.5 <u>Compensation</u>

Riemann & Company a/s maintain in force a "no fault" compensation insurance indemnity in accordance with the current version of the ABPI Guidelines on Clinical Trials: "Compensation for Medical Induced Injury ". In the event that the compensation on a "no fault basis" is unacceptable to the claimant, the policy will, subject to its terms, conditions and limitations, respond to an action for legal liability arising out of this clinical trial.

4.6 <u>Investigator's Responsibilities</u>

i. <u>Good Clinical Practice</u>

It is the responsibility of each and every investigator to ensure that this study is carried out in accordance with this protocol in respect of ethical, legal and technical aspects and conforming to both the Declaration of Helsinki and European (CPMP) Guidelines on Good Clinical Practice for Trials on Medicinal Products (see Appendix 6) and also the European standard, EN 540: Clinical investigation of medicinal devices for human subjects (see Appendix 7). In this context, the investigator shall arrange for the retention of patient identification codes for at least 15 years after completion or discontinuation of the trial. The sponsor will render all support necessary to assist the investigator in discharging this responsibility.

ii. <u>Replacement of Investigator</u>

In the event of the investigator being unable to continue the study, another responsible person will be designated investigator and documentation testifying this will be submitted to the study monitor within 10 days. The new investigator must be appropriately qualified and approved by the sponsor and the local ethics committee before the study can be continued.

iii. <u>Study Report</u>

The investigator will submit a summary trial report within approximately 1 month of completion of the study. This report will include:

1. Details of the investigative procedures involved

2. The number of patients entered, completed and withdrawn from the study.

3. Deviations from the study protocol on a general basis and for individual patients with explanations.

4. Explanations for each patient withdrawn from the study

5. Methodology and normal ranges for laboratory investigations (where appropriate)

6. Summary of demographic details for each treatment group, e.g. sex, age etc.

7. Summary of the safety and tolerance data, including:

   Details of all ADEs including any follow-up. Case histories of all serious ADEs or ADEs leading to withdrawal.

8. If appropriate, details of any statistical analysis carried out by the investigators, and a summary of efficacy data including clinical observations.

9. Conclusions.

4.7 Curriculum Vitae

In accordance with the international standards, and Good Clinical Research Practice, a signed copy of the curriculum vitae of each investigator and co-worker will be provided to the study monitor.

4.8 Case Report Form

The investigator is required to prepare and maintain adequate and accurate case records which have been designed specifically for this study, and to record all observations and other data pertinent to the clinical investigation. All record forms should be completed in their entirety in a neat, legible manner to ensure accurate interpretation of data. **Blue or Black-ball-point pen** should be used to ensure the clarity of the reproduced copy of all case report forms.

The CPMP Guidelines on Good Clinical Practice for Trials on Medicinal Products in the European Community require that the investigator shall arrange for the retention of the patient identification codes for at least 15 years after the completion or discontinuation of the trial. Patient files and other source data shall also be kept by the Medical Entomology Centre for a period of not less than 15 years.

4.9 Monitoring of the Study

At regular intervals during the study, the study centre may be visited by a representative of the monitoring team of the Sponsor.

4.10 Quality Assurance

In accordance with the Good Clinical Practice Guidelines and recommendations, the Sponsor may undertake a quality assurance audit of the clinical trial and related documentation during the course of this trial.

The purpose of the QA audits is to check on the monitoring of studies and to try to reduce inconsistencies such as transcription errors and errors in logical sequencing. It is important for investigators to maintain an accurate set of patient notes. This is essential material for auditing purposes. At any stage during the trial, the investigator has the responsibility to make all the data available to the sponsor and/or the relevant authority

(where required) for auditing purposes. Such audits will at all times be conducted with national, legal and ethical requirements.

4.11 Protocol Appendices

It is specified that the appendices attached to this protocol, and referred to in the main text of this protocol, form an integral part of the protocol.

4.12 Protocol Amendments

No changes or amendments to this protocol can be made by the investigator or by Riemann & Company a/s unless such change(s) or amendment(s) have been fully discussed and agreed upon by both the investigator and Riemann & Company a/s. Any change or amendment agreed upon will be recorded in writing, the written amendment will be signed by the investigator and by Riemann & Company a/s and the signed amendment will be appended to this protocol. A copy of any such changes must be submitted to the Research Ethics Committee for approval before they may be acted upon.

4.13 Publication Policy

Publication results of the study will not take place without prior discussion with Riemann & Company a/s. The company will be allowed sufficient time to analyse these results and provide written agreement to publication. In turn Riemann & Company a/s agree that permission to publish will not be unreasonably withheld. Riemann & Company a/s reserve the right to use the results and reports of this study for their own commercial purposes.

5. Investigator's Agreement

We have read this Riemann & Company a/s approved protocol, number CT RL02, dated – July 1999 entitled "A randomised, parallel group clinical trial to assess the efficacy, safety and acceptability of a surfactant based lotion.

We agree to conduct the study according to this protocol and to comply with its obligations, subject to ethical and safety considerations.

We understand that should we be in the breach of any of the terms of this protocol, or if we are negligent, that Riemann & Company a/s, would not be held responsible for any resulting losses, damages, costs and expenses of whatever kind made by or on behalf of a volunteer.

Principal Investigator :________________________

Co-investigators :________________________

:________________________

[Clinical research Manager] :______________________

[Technical Director] :______________________

Should the decision be made by Riemann & Company a/s to terminate the study at any time, such decision will be communicated to the investigator in writing, and appropriate agreements will be agreed upon and specified in writing. Conversely, should the investigator decide to withdraw from execution of the study he/she will communicate immediately such decision in writing to the Sponsor.

APPENDIX 1

MEDICAL ENTOMOLOGY CENTRE

Telephones: [number no longer functional]

Fax: [number no longer functional]

Address line 1
Address line 2
Cambridge

To be dated

Dear Headteacher

The Medical Entomology Centre, in conjunction with Dr N Irish, Consultant in Communicable Disease Control with Cambridgeshire Health Authority, is embarking on a second study to evaluate a potential new way of delivering head louse treatment, which it is hoped will be just as effective as those currently available.

We are planning to recruit 120 patients with head lice for the study and wonder if you would be prepared to distribute information letters to families via your school. School will not be involved administratively in any other way.

If you would like any further information please call Mr Ian Burgess or Mrs Christine Brown at the Medical Entomology Centre on [number no longer functional]. We would appreciate a reply as soon as possible as we hope to begin the study immediately.

Yours sincerely

Mrs Christine Brown
Medical Entomology Nursing Sister

MEDICAL ENTOMOLOGY CENTRE

Telephones: [number no longer functional]
Fax: [number no longer functional]

Address line 1
Address line 2
Cambridge

To be dated

Dear Dr

The Medical Entomology Centre, in conjunction with Dr N Irish, Consultant in Communicable Disease Control with Cambridgeshire Health Authority, is embarking on a second study to evaluate a potential new way of delivering head louse treatment, which we hope will be just as effective as those currently available.

We are planning to recruit 120 patients with head lice for the study and since we are aware that this infection often takes up your surgery time and budget, wondered if you might be prepared to direct patients to the study for treatment and follow up.

It is hoped to begin the study in immediately. We will of course inform you if any of your patients join the programme. Ethical approval has been granted by [name of ethics committee]. I enclose a patient information sheet. Should you require any further details please contact Mr Ian Burgess/Mrs Christine Brown at the Medical Entomology Centre.

Yours sincerely

Mrs Christine Brown
<u>Medical Entomology Nursing Sister</u>

Enc

MEDICAL ENTOMOLOGY CENTRE

Telephones: [number no longer functional]
Fax: [number no longer functional]

Address line 1
Address line 2
Cambridge

Dear Parent or Guardian

To be dated

The Medical Entomology Centre is currently involved in carrying out a field study to assess a new product for the treatment of head lice that follows on from one conducted in 1998.

The new product has been developed in Denmark and is based on a wetting agent widely employed in the cosmetic industry. The product can be applied as easily as any currently available lotions used in the treatment of head louse infection but it does not incorporate an insecticide.

Volunteers will be randomized into one of two groups:

Group 1 - The lotion will be applied to dry hair then dried with a hairdryer. It will be washed off with clear water after a minimum of 8 hours.

Group 2 - The lotion will be applied to dry hair then dried with a hairdryer. It will be washed off with clear water after 2 hours. This treatment will be repeated 7 days later.

If you or anyone in your family has a head louse infection over the next few months perhaps you would consider taking part in this study which will commence immediately. If you are interested in joining the study and have not used a head lice treatment during the previous 2 weeks please contact Ian Burgess or myself at the above address as soon as you discover the infection and arrangements will be made to visit you at home. If you contact us by telephone out of office hours leave your name, address, and telephone number (including the STD code) on the answering machine and you will be contacted as soon as possible.

Please do not remove the lice as the most important criterion for this study is the presence of live lice.

Yours sincerely

Mrs Christine Brown
Medical Entomology Nursing Sister

Media

A clinical study is being carried out in the Cambridgeshire area in an attempt to combat head lice. Scientists at the Medical Entomology Centre in Cambridge are appealing for volunteers who suffer from head lice to contact them before they seek alternative treatment. Nursing sister Christine Brown, said "We are looking for 120 volunteers in this area who are willing to give a new lotion a try. The product has been developed by a pharmaceutical company in Denmark and does not contain any insecticides". She explained the need for a new treatment has been heightened by the fact that lice are developing a UK-wide resistance to existing products and there is a move by scientists to minimise the use of insecticides. Volunteers are needed to take part in the 14 day clinical trial. An initial study has already shown that the product has potential in this field.

Mrs Brown added "Although we cannot guarantee this product will rid people of their head lice, volunteers will benefit because the treatment is free and they will get lots of professional one to one help. We are aware that many people are having problems ridding themselves and their families of this infection".

The trial lotion contains ingredients which have been widely used in the cosmetics industry for some time. It is applied to dry hair like several other louse lotions. This area has been chosen for the study because of its close proximity to Cambridge where the research work is being carried out. **CONTACT**; [number no longer functional] if you are interested in joining the study. Volunteers will be asked various questions over the phone and a personal visit can usually be made within 24 hours.

FACT FILE

* Head lice are small insects, roughly the size of a sesame seed, and usually grey or brown in colour.
* They cannot fly, jump or swim, but spread by clambering from head to head.
* They are not fussy about the length of hair or how clean it is.
* Anyone can catch them, but children are most commonly affected. It is estimated one in ten primary school children will be affected every year.
* The lice feed by sucking blood through the scalp of their host.
* Female lice lay eggs which are glued to the base of the hair. The nits which everyone talks most about are in fact previously hatched eggs and as such are harmless.

APPENDIX 2

INFORMATION DOCUMENT

1. The company sponsoring the study is: Riemann & Co a/s, Hilleroed, Denmark.

2. The main applicant is: Ian F Burgess, Medical Entomology Centre.

3. The contact physician is: Dr N Irish, Cambridgeshire HealthAuthority.

4. The Research Team members are: Christine M Brown, Medical Entomology Centre
   Elizabeth Basham, Medical Entomology Centre

5. If assistance or advice is required during the 14 days of the study please use the following contact telephone: [number no longer functional] (Monday to Friday 0900 to 1700 hours). At all other times an answering machine will take a message. Please leave your name and telephone number, someone will contact you as soon as possible. If a medical problem arises please contact your own General Practitioner, who will be informed that you are participating in this study, or call the Medical Contact, Dr N Irish on [number no longer functional].

6. This study has been subject to review by an independent ethics research committee.

7. Volunteers will be randomized into one of two groups:
   Group 1 - The lotion will be applied to dry hair then dried with a hairdryer. It will be washed off with clear water after a minimum of 8 hours.

   Group 2 - The lotion will be applied to dry hair then dried with a hairdryer. It will be washed off with clear water after 2 hours. This treatment will be repeated 7 days later.

8. All patients should inform the Medical Entomology Centre or the Medical Contact of any of the following:

   a) Any illness arising during the course of the study

   b) Any abnormal occurrences thought to be due to either of the treatments

   c) Any intention to discontinue the study

9. If you are unable to keep an appointment for a visit by one of the investigators please telephone the Medical Entomology Centre [number no longer functional] to make alternative arrangements as soon as possible.

10. As required by law all documentation pertaining to this study will be held by the Medical Entomology Centre for a period of 15 years. Personal details of those taking part will remain confidential.

VOLUNTEER AGREEMENT AND CONSENT FORM

1. I, the undersigned, voluntarily agree to participate in a field study using a head louse treatment product.

2. I have been given a full explanation by the supervising professional, of the nature, purpose and likely duration of the study and what I will be expected to do and I have been advised about any discomfort and possible ill-effects on my health or well-being which they believe may result. The information document given to me is attached.

3. I have been given the opportunity to question the supervising professional on all aspects of the study and have understood the advice and information given as a result.

4. I agree to the supervising professional contacting my General Practitioner to make known my participation in the study and I authorise my doctor to disclose details of any relevant medical or drug history, in confidence.

5. I agree to comply with any instruction given during the study and to co-operate faithfully with the supervising professionals and to tell them immediately if I suffer any deterioration of any kind in my health or well-being or any unexpected or unusual symptoms however they may have arisen.

6. I agree that I will not seek to restrict the use to which the results of the study may be put and, in particular, I accept that they may be disclosed to regulatory authorities for medicines in the UK and elsewhere.

7. I understand that I am free to withdraw from the study at any time without needing to justify my decision.

CHILDREN'S INFORMATION SHEET

A TEST ON A NEW LOTION TO KILL HEAD LICE

We have been asked to test a new lotion that kills head lice. If you have head lice maybe you would like to help us to see how well it works.

We will visit you at home and comb your hair to see if there are any lice. There are two ways the lotion can be used.

1. The lotion is put on dry hair. It is then dried with a hair dryer. After 8 hours, or the next day, the lotion is washed off with warm water.
2. The lotion is put on dry hair. It is then dried with a hair dryer. After 2 hours the lotion is washed off with warm water. This will be done again after one week.

You will be given your own small bottle of shampoo and asked to wash your hair 3 days later.

We need everyone to help the study for 14 days. In these 2 weeks other people will visit you at home to see if there are any lice left. They will do this four times by combing your hair with a plastic louse comb.

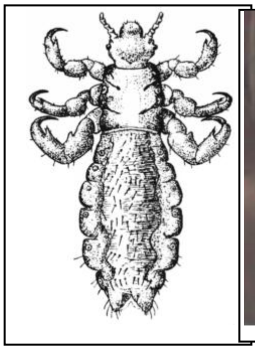

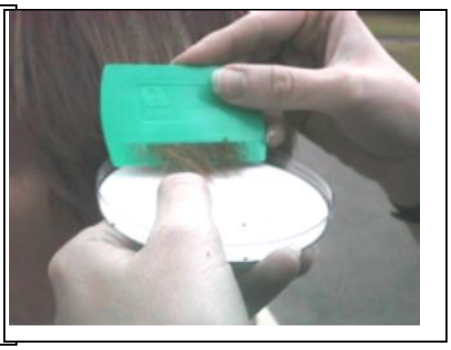

MEDICAL ENTOMOLOGY CENTRE

Consent by a Patient to the participation in a Clinical Trial of a Therapeutic Procedure/Drug or other Clinical Research.

I ....

of ....

hereby fully and freely consent to participate in the investigation entitled:

A randomised, parallel group clinical trial to evaluate the efficacy, safety and acceptability of a surfactant based lotion for the treatment of head lice.

I understand and acknowledge that the trial is designed to add to medical knowledge. I note that I may withdraw my consent at any stage in the Investigation and I acknowledge that the purpose of the Investigation, the risks involved from drugs or other procedures, and the nature and purpose of such procedures have been explained to me by:

....

and that I have had an opportunity to discuss these matters with him/her.

I consent to the disclosure of personal information (under the Data Protection Act) in relation to this Research Project.

I have received a written explanation of these matters, a copy of which is attached to this form.

Signed .... Date ....

WITNESS to the fact that he/she has read the document and freely given his/her consent.

Address:...

Occupation:..

Signed .... Date ....
(Witness **must not** be a member of project team)

I confirm that I have explained to the patient/volunteer the nature and effect of these procedures.

Signed .... Date ....

Name of Researcher responsible for Investigation..
(Block capitals)

VOLUNTEER AGREEMENT AND CONSENT FORM

1. I, the undersigned, voluntarily agree to my child/children, named in this document, participating in a field study using a head louse treatment product.

2. I have been given a full explanation by the supervising professional, of the nature, purpose and likely duration of the study and what my child/children will be expected to do and I have been advised about any discomfort and possible ill-effects on my child / children's health or well-being which they believe may result. The information document given to me is attached.

3. I have been given the opportunity to question the supervising professional on all aspects of the study and have understood the advice and information given as a result.

4. I agree to the supervising professional contacting my child/children's general practitioner to make known their participation in the study and I authorise my child/children's general practitioner to disclose details of their relevant medical or drug history, in confidence.

5. I agree to comply with any instruction given during the study and to co-operate faithfully with the supervising professionals and to tell them immediately if my child/children suffers any deterioration of any kind in his/her health or well-being or any unexpected or unusual symptoms however they may have arisen.

6. I agree that I will not seek to restrict the use to which the results of the study may be put and, in particular, I accept that they may be disclosed to regulatory authorities for medicines in the UK and elsewhere.

7. I understand that I am free to withdraw my child/children from the study at any time without needing to justify my decision.

Medical Entomology Centre

Consent by Legal Guardian of a Minor to the participation by the Minor in a Clinical Trial of a Therapeutic Procedure.

I ....

of ....

being the legal guardian of ……………………………………………………………………………………………….

of age .... (subsequently referred to as child) hereby give my permission fully and freely for the child to participate in the controlled clinical trial entitled:

A randomised, parallel group clinical trial to assess the efficacy, safety and acceptability of a surfactant based lotion for the treatment of head lice.

I understand and acknowledge that the trial is designed to promote medical knowledge and that my child may or may not be receiving the most effective available treatment in the first instance.

I note that I may withdraw my consent at any stage in the investigation and I acknowledge that the purpose of the trial, has been explained to me by:

....

and that I had an opportunity to discuss these matters with him/her.

I have received a written explanation of these matters, a copy of which is attached to this form.

Signed .... Date ....

WITNESS to guardian's signature and to the fact that he/she has read the document and freely given his/her consent.

Signed .... Date ....

(Witness **must not** be a member of project team)

I confirm that I have explained to the legal guardian of the child the nature and effect of these procedures.

Signed .... Date ....

Name of Researcher responsible for Investigation ....
(Block capitals)

APPENDIX 3

MEDICAL ENTOMOLOGY CENTRE

Telephones: [number no longer functional]
Fax: [number no longer functional]

Address line 1
Address line 2
Cambridge

To be dated

Dear Dr

The Local Research Ethics Committee has asked us to tell you that the person named below, who is registered with you has agreed to take part in our research project as a volunteer.

Please keep this as a permanent record of their involvement. We shall also inform you later if there are any abnormal findings or possible adverse effects noted by us.

Name of subject	Date of birth	Date(s) of participation in study

Title of Project	Study Reference Number

A randomised, clinical trial to assess the efficacy, safety and acceptability of a surfactant based lotion for the treatment of head lice.

Yours sincerely

Mrs Christine Brown
<u>Medical Entomology Nursing Sister</u>
